# Evolutionary Convergence and Nitrogen Metabolism in *Blattabacterium* strain Bge, Primary Endosymbiont of the Cockroach *Blattella germanica*

**DOI:** 10.1371/journal.pgen.1000721

**Published:** 2009-11-13

**Authors:** Maria J. López-Sánchez, Alexander Neef, Juli Peretó, Rafael Patiño-Navarrete, Miguel Pignatelli, Amparo Latorre, Andrés Moya

**Affiliations:** 1 Institut Cavanilles de Biodiversitat i Biologia Evolutiva, Universitat de València, València, Spain; 2 CIBER en Epidemiología y Salud Pública (CIBEResp), Barcelona, Spain; 3 Departament de Bioquímica i Biologia Molecular, Universitat de València, València, Spain; 4 Centro Superior de Investigación en Salud Pública (CSISP), València, Spain; 5 Departament de Genètica, Universitat de València, València, Spain; Vanderbilt University, United States of America

## Abstract

Bacterial endosymbionts of insects play a central role in upgrading the diet of their hosts. In certain cases, such as aphids and tsetse flies, endosymbionts complement the metabolic capacity of hosts living on nutrient-deficient diets, while the bacteria harbored by omnivorous carpenter ants are involved in nitrogen recycling. In this study, we describe the genome sequence and inferred metabolism of *Blattabacterium* strain Bge, the primary Flavobacteria endosymbiont of the omnivorous German cockroach *Blattella germanica*. Through comparative genomics with other insect endosymbionts and free-living Flavobacteria we reveal that *Blattabacterium* strain Bge shares the same distribution of functional gene categories only with *Blochmannia* strains, the primary Gamma-Proteobacteria endosymbiont of carpenter ants. This is a remarkable example of evolutionary convergence during the symbiotic process, involving very distant phylogenetic bacterial taxa within hosts feeding on similar diets. Despite this similarity, different nitrogen economy strategies have emerged in each case. Both bacterial endosymbionts code for urease but display different metabolic functions: *Blochmannia* strains produce ammonia from dietary urea and then use it as a source of nitrogen, whereas *Blattabacterium* strain Bge codes for the complete urea cycle that, in combination with urease, produces ammonia as an end product. Not only does the cockroach endosymbiont play an essential role in nutrient supply to the host, but also in the catabolic use of amino acids and nitrogen excretion, as strongly suggested by the stoichiometric analysis of the inferred metabolic network. Here, we explain the metabolic reasons underlying the enigmatic return of cockroaches to the ancestral ammonotelic state.

## Author Summary

Bacterial endosymbionts from insects are subjected to a process of genome reduction from the moment they interact with their host, especially when the symbiosis is strict (the partners live together permanently) and the endosymbiont is maternally inherited. The type of genes that are retained correlates with specific metabolic host requirements. Here, we report the genome sequence of *Blattabacterium* strain Bge, the primary endosymbiont of the German cockroach *B. germanica*. Cockroaches are omnivorous insects and *Blattabacterium* cooperates with their metabolism, not only with essential nutrient metabolism but also through an efficient use of amino acids and the nitrogen excretion by the combination of a urea cycle and urease activity. The repertoires of functions that are maintained in *Blattabacterium* are similar to those already observed in *Blochmannia* spp., the primary endosymbiont of carpenter ants, also an omnivorous insect. This constitutes a nice example of evolutionary convergence of two endosymbionts belonging to very different bacterial phyla that have evolved a similar repertoire of functions according to the host. However, the current set of genes and, more importantly, those that were lost in the process of genome reduction in both endosymbiont lineages have also contributed to a different involvement of *Blattabacterium* and *Blochmannia* in nitrogen metabolism.

## Introduction

In 1887, Blochmann first described symbiotic bacteria in the fatty tissue of blattids [1]. Later, Buchner [2] suggested that symbionts are involved in the decomposition of metabolic end-products from the insect host. A classic example is the cockroach. Several pioneering studies correlated the presence of cockroach endosymbionts with the metabolism of sulfate and amino acids [3],[4]. These endosymbionts were classified as a genus *Blattabacterium* [4], belonging to the class Flavobacteria in the phylum Bacteroidetes [5] and they live in specialized cells in the host’s abdominal fat body. Apart from cockroaches, they were only found in the primitive termite *Mastotermes darwiniensis* [6]. Phylogenetic analyses for the *Blattabacterium*-cockroach symbiosis supported the hypothesis of co-evolution between symbionts and hosts dating back to an ancient feature of more than 140 million years ago [7],[8]. Recently, genome sizes of the *Blattabacterium* symbionts of three cockroach species, *B. germanica*, *Periplaneta americana*, and *Blatta orientalis* were determined by pulsed field gel electrophoresis as approximately 650±15 kb [9]. Similarly, the authors demonstrated the sole presence of *Blattabacterium* strains in the fat body of those cockroach species by rRNA-targeting techniques. Phylogenetic analyses based on 16S rDNA also confirmed the affiliation of these endosymbionts to the class Flavobacteria [9]. Therefore, they are phylogenetically quite distinct from the majority of intensively studied insect endosymbionts that belong to the phylum Proteobacteria, mainly class Gamma-Proteobacteria. Recently, the highly reduced genome of “*Candidatus* Sulcia muelleri” (from now *S. muelleri*), an insect endosymbiont belonging to the class Flavobacteria has been also completely sequenced [10].

Primary endosymbionts such as *Buchnera aphidicola* or *Wigglesworthia glossinidia* complement the metabolic capacity of aphids or tsetse flies, respectively that feed on different nutrient-deficient diets [11]. There are also examples of metabolic complementation between two co-primary endosymbionts and their hosts. This is the case of *S. muelleri*, living in the sharpshooter *Homalodisca vitripennis*, which coexists with another Gamma-Proteobacteria endosymbiont, “*Candidatus* Baumannia cicadellinicola” (hereafter *B. cicadellinicola*). Both have developed a metabolic complementation to supply the host with the nutrients lacking in the limited xylem diet [12]. Another example is the case of *B. aphidicola* and “*Candidatus* Serratia symbiotica”, co-primary endosymbionts of the cedar aphid *Cinara cedri* that complement each other in the provision of essential nutrients [13],[14].

Omnivorous insects also harbor endosymbionts. It is the case, for example, of ants of the genus *Camponotus* and their primary endosymbionts, the Gamma-Proteobacteria “*Candidatus* Blochmannia floridanus” [15] and “*Candidatus* Blochmannia pennsylvanicus” [16] (from now *B. floridanus* and *B. pennsylvanicus*, respectively). In this association endosymbionts play an important role in nitrogen recycling [17].

Evolutionary convergences are generally considered as evidence of evolutionary adaptation. The study of endosymbiont evolution could provide examples of evolutionary convergences if we were able to show that very distant phylogenetic groups present similar functional repertoires and metabolic capabilities when they have evolved endosymbiosis in organisms having similar feeding behaviors. This may be the case of *Blochmannia* (a gamma-proteobacterium) and *Blattabacterium* (a flavobacterium) that have independently evolved in carpenter ants and cockroaches, two omnivorous insects.

In this study, we determine the genome sequence of an endosymbiotic flavobacterium, *Blattabacterium* strain Bge, primary endosymbiont of the German cockroach *B. germanica*. We have also inferred the metabolism to try to understand why cockroaches excrete ammonia, instead of being uricotelic like other terrestrial invertebrates, thus breaking the so-called “Needham's rule” [18], a question that has puzzled physiologists for a long time. Finally, we compare the inferred metabolism with the corresponding one of *B. floridanus*, the primary endosymbiont involved in nitrogen recycling in the carpenter ant *Camponotus floridanus*, an insect that has also a complex diet.

## Results/Discussion

### Genome of *Blattabacterium* strain Bge

The general features of the genome of *Blattabacterium* strain Bge (CP001487) and their comparison with those of other selected bacteria are shown in Table 1. The size of the circular chromosome is 637 kb, and the G+C content is 27.1%. Only 23.4 kb are not-coding and they are distributed in 480 intergenic regions with an average length of 49 bp.

**Table 1:** Comparative genomics of *Blattabacterium* strain Bge with those of *S. muelleri* (NC_010118), *Flavobacterium psychrophilum* (NC_009613), *B. floridanus* (NC_005061), *B. pennsylvanicus* (NC_007292), *B. aphidicola* BAp (NC_002528), and *B. aphidicola* BCc (NC_008513).

	*Blattabacterium* strain Bge	*S. muelleri*	*F. psychrophilum* a	*B. floridanus*	*B. pennsylvanicus*	*B. aphidicola* BAp	*B. aphidicola* BCc
Phylum, class	Bacteroidetes, Flavobacteria			Proteobacteria, Gamma-Proteobacteria			
Genome size (bp)	636,850	245,530	2,865,395	705,557	791,654	640,681	422,434
Plasmids	-	-	1	-	-	-	1
Plasmid size (bp)	-	-	3,407	-	-	-	6,054
Chromosome size (bp)	636,850	245,530	2,861,988	705,557	791,654		416,380
G+C content (%)	27.1	22.4	32.5	27.4	29.6	26.3	20.1
Total number of genes	627	263	2503	636	658	607	397
CDSs	586	228	2416	583	610	564	357
rRNAs	3	3	18	3	3	3	3
tRNAs	34	31	67	37	39	32	31
Other RNAs	3	1	2	2	2	1	3
Pseudogenes	1	-	20	4	4	1	3
Overall coding region (%)	96.3	96.1	89.2	85.0	79.3	89.1	87.7
Coding region CDS (%)	95.0	91.9	84.5	83.1	76.7	86.9	85.0
CDSs average length (bp)	1,034	996	1,004	1,007	995	988	992

a Free-living species

The overall coding density (96.3%) is the highest among insect endosymbionts known to date, indicating a highly compact genome. It is surprisingly higher than the most reduced insect endosymbiont “*Candidatus* Carsonella ruddii” (93.4%) [19]. In addition, 1.5 kb correspond to 139 overlapping regions with an average length of 11 bp. Of these overlaps, 94 (67.6%) are between genes on the same strand and 1 to 70 bp long. The other 45 cases (32.4%) involve two genes on opposite strands and are between 2 and 50 bp long. Of these, only in one case the two genes overlap with their start regions, whereas in the rest the overlap is in the terminal region of the genes. On the other hand, in “*Ca* Carsonella ruddii” 92% of the 126 overlaps are in tandem orientation, and thus on the same strand, and only five cases are between opposite strands, involving the termini and starts of the overlapping genes.

Assembly of the pyrosequencing data gave highly reliable contigs that combined with the data from Sanger sequencing resulting in a single contig, representing the entire genome. Probably due to the formation of a secondary structure, only a 33 bp stretch in an intergenic region upstream of the GroEL gene was not covered by pyrosequencing data but only by Sanger reads. Furthermore, annotation of the ORFs allowed a clear assignation of protein functions even in cases with only weak similarities with existing database entries. Not a single case of a possible host gene incorporated in the symbiont genome was found. Neither had we found coding sequences affiliated with *Blattabacterium* strain Bge outside the genome that could have been assigned to the host genome.

A total of 627 putative genes have been assigned (Figure S1), 586 of which are protein coding genes (CDS), 40 are RNA-specifying genes (34 tRNAs, 3 rRNAs located in a single operon, one tmRNA, and the RNA components of RNase P and the Signal recognition particle). The only pseudogene found corresponds to the protein component of RNase P. This gene coding for 118 amino acids is disrupted by an in-frame stop codon at amino acid position 53. The RNase P proteins of the free-living *F. psychrophilum* [20], *Flavobacterium johnsoniae* (http://genome.jgi-psf.org/flajo/flajo.info.html) and *Gramella forsetii* [21] contain a lysine residue at that position. Therefore, it is possible that the stop codon has been generated by an A–T point mutation in position 157 of the nucleotide sequence. Despite this mutation, the RNase P could be functional as it has been described that *in vitro* the RNA component can act enzymatically without a functional protein component [22]. Regarding the coding genes, it is interesting that, despite the compactness of the genome, there are eight gene duplicates: *miaB*, *rodA*, *serC*, *lpdA*, *ppiC*, *argD*, *hemD*, and *uvrD*.

No specific sequence of the origin of replication (*oriC*), such as dnaA boxes, was found in the genome [23]. Likewise *dnaA*, which codes for the protein that initiates replication by binding to such sequences, was also absent. Thus, the putative origin of replication was determined by GC skew analysis. The transitional region where the GC skew changes from negative to positive one (Figure S2) showed the position of replication origin to be in the gene *dapB*. It is worth mentioning that neither *dnaA* nor any of the genes normally adjacent to the replication site in bacteria (*dnaN*, *hemE*, *gidA*, *hemE*, and *parA*) have been found in this genome. However, *Blattabacterium* strain Bge, has retained *recA*, which could trigger replication by an alternative mechanism [15],[23].

### Functional analysis of the predicted protein-coding genes

**Figure 1:**
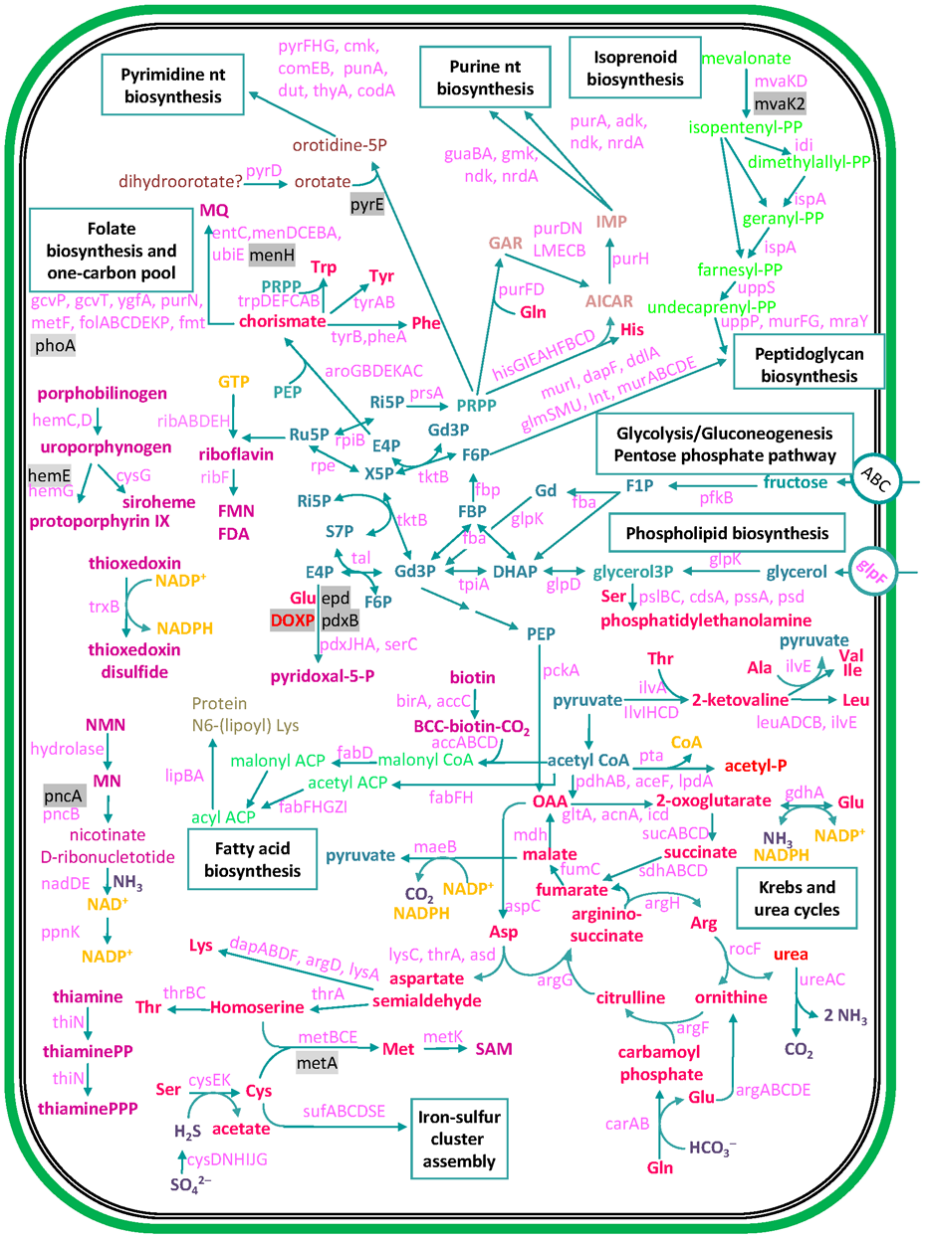
An integrated view of the predicted metabolism and transport in *Blattabacterium* strain Bge.

We have inferred the metabolism of *Blattabacterium* strain Bge from its complete genome (Figure 1). *Blattabacterium* strain Bge possesses a limited capacity for nutrient uptake with only one ABC-type transport system, which may be specialized in fructose transport because this bacterium, contrary to the other sequenced endosymbionts, seems unable to use glucose as a nutrient. On the other hand, *Blattabacterium* strain Bge also codes for a glycerol uptake facilitator that enables transport of solutes, such as O_2_, CO_2_, NH_3_, glycerol, urea, and water. Therefore, it is possible that *Blattabacterium* strain Bge obtains carbon from glycerol as a supplementary source.

A sodium/drug antiporter, NorM, is also encoded by this genome. This system of efflux drug transport is common among enterobacteria but not among flavobacteria. In this group it is only known for the free-living bacteria *F. psychrophilum* and *G. forsetii*. This system can act as a multidrug transport as well as transporting oligosaccharidyl lipids and polysaccharide compounds.

There is an array of metal ion homeostasis transporters. In *Blattabacterium* strain Bge, there is a Trk transport system, a uniporter of the monovalent potassium cation, which requires a proton motive force and ATP in order to function. Only *W. glossinidia* has a similar transport system, although the encoded subunits differ: trkA and trkB in *Blattabacterium*; trkA and trkH in *W. glossinidia*. Other solutes are also transported by symport systems. *Blattabacterium* strain Bge is able to uptake glutamate and aspartate via a proton symporter. Both metabolites play an important role in the metabolism of this bacterium (see below). A phosphate/sodium symporter is also present.

Regarding electron transport, the encoded NADH-dehydrogenase (*ndh*) oxidizes NADH without proton translocation. There is also a succinate dehydrogenase (*sdh*ABD). Electrons are transferred to a membrane-bound menaquinone (MQ) and a molybdenum-oxidoreductase, which accepts electrons from the MQ. With these elements, a proton motive force can be generated.

*Blattabacterium* strain Bge seems to be able to reduce intracellular sulfate to sulfite. A number of genes required for sulfur assimilation present in the genome, include those encoding for the two subunits of the sulfate adenylyltransferase, *cysN* and *cysD*, the adenosine phosphosulfate (APS) reductase *cys*H and the sulfite reductase proteins *cysI,J*. There is a missing step for the conversion of adenosine-5′-phosphosulfate (APS) into 3′-phospho adenosine-5′-phosphosulfate (PAPS). The generated sulfite is reduced to sulfide further on and assimilated into the sulfur-containing amino acids L-cysteine and L-methionine.

*Blattabacterium* strain Bge is able to synthesize its own cell wall and plasma membrane. However, it has lost the entire pathway required for lipopolysacharide (LPS) biosynthesis, like all sequenced *Buchnera* strains and *B. cicadenillicola*. This property explains why *Blattabacterium* strain Bge, similarly to these bacteria, are surrounded by a host vacuolar membrane, as shown in the electron-microscopy images (Figure S3).

Regarding amino acid biosynthesis, *Blattabacterium* strain Bge has the genes encoding biosynthetic enzymes needed to synthesize 10 essential (His, Trp, Phe, Leu, Ile, Val, Lys, Thr, Arg, and Met) and 7 nonessential (Gly, Tyr, Cys, Ser, Glu, Asp, and Ala) amino acids. Thus, the endosymbiont metabolism relies on Pro, Gln and Asn supplied by the host. Also present is the complete machinery to synthesize nucleotides, fatty acids, and the cofactors folic acid, lipoic acid, FAD, NAD, pyridoxine, and riboflavin. Finally, genes encoding enzymes for the synthesis of siroheme and menaquinone were also identified.

With respect to the metabolism of carbohydrates, genome analysis of *Blattabacterium* strain Bge indicates the presence of a truncated glycolysis pathway, since the genes that encode for phosphofructokinase (*pfk*A) and pyruvate kinase (*py*k) are missing, as well as any sugar phosphorylating system except for fructose. Therefore, the pathway begins with fructose-1 phosphate and continues with the canonical enzymatic steps until the synthesis of phosphoenolpyruvate (PEP). Given the lack of pyruvate kinase genes, *Blattabacterium* strain Bge must produce pyruvate via the malic enzyme (NADP^+^-dependent malate dehydrogenase). Additionally, a complete non-oxidative pentose phosphate pathway is encoded in *Blattabacterium* strain Bge. As it is the case with *Wigglesworthia*, the glycolytic enzymes seem to be involved in gluconeogenesis rather than glycolysis complementing the non-oxidative pentose phosphate pathway [24].

In summary, although *Blattabacterium* strain Bge genome shows a strong reduction in gene number in all the functional categories, compared to their free-living relatives (see below), the core of essential functions and pathways is particularly well preserved.

### Comparative analysis and functional convergence

**Figure 2:**
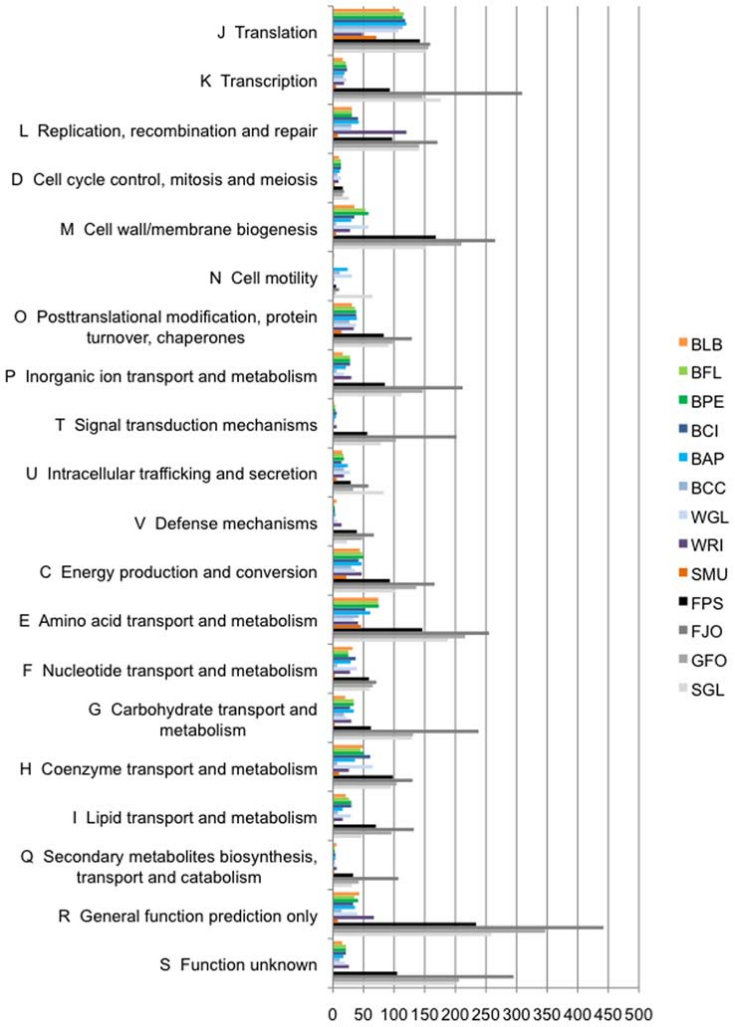
Absolute number of genes for the different COG categories. Species abbreviations are as follows: BLB, *Blattabacterium* strain Bge; BFL, *B. floridanus*; BPE, *B. pennsylvanicus*; BCI, *B. cicadellinicola* (NC_007984); BAP, *B. aphidicola* Bap; BCC, *B. aphidicola* BCc; WGL, *W. glossinidia* (NC_003425); WRI, *Wolbachia* sp. wRi (NC_012416); SMU, *S. muelleri*; FPS, *F. psychrophilum*; FJO, *F. johnsoniae* (NC_009441); GFO, *G. forsetii* (NC_008571); SGL, *Sodalis glossinidius* (NC_007712).

**Figure 3:**
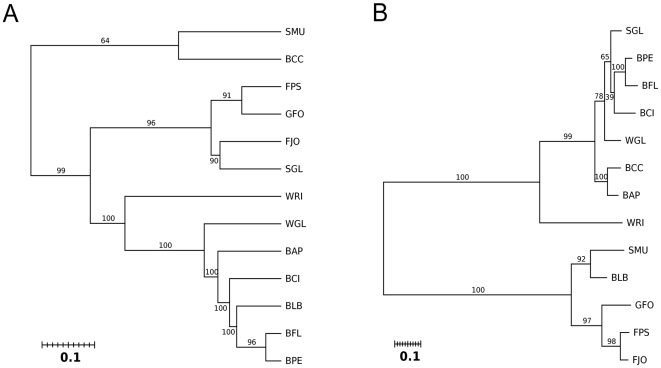
Functional closeness and phylogenetic relationship of endosymbionts. (A) Dendrogram obtained by a linkage clustering method from the matrix of Kulczynski distances between species for the observed distribution of COG categories (Figure 2, Table 2). In all cases, except one, the null hypothesis of getting by chance the corresponding cluster was rejected (bootstrap values were equal or higher than 90%). (B) 16S rDNA maximum likelihood phylogenetic tree, with bootstrap values (%) based on 1000 replicates, of the thirteen compared bacterial species. The methods used to derive the Kulczynski distance, the dendrogram and the phylogenetic tree are detailed in Materials and Methods. Species abbreviations as in Figure 2. 16S rDNA gene NCBI-GeneID: BLB, 99077774; BFL, 1499754; BPE, 3563224; BCI, 4056264; BAP, 7262504; BCC, 4441000; SMU, 5797390; WGL, 1257559; WRI, 7669911; FPS, 5300282; FJO, 5092512; GFO, 4652227; SGL, 3866283.

The protein genes of *Blattabacterium* strain Bge were classified according to COG categories (Figure 2, Table 2). This distribution was compared with those of twelve selected bacteria: four Flavobacteria, which included three free-living species (*F. psychrophilum*, *F. johnsoniae* and *G. forsetii*) and the endosymbiont *S. muelleri*, and eight Proteobacteria endosymbionts, seven Gamma-Proteobacteria (*B. floridanus*, *B. pennsylvanicus*, *B. cicadellinicola*, *B. aphidicola* Aps, *B. aphidicola* Cce, *S. glossinidius*, and *W. glossinidia*) and one Alfa-Proteobacterium (*Wolbachia* sp. from *Drosophila simulans*). Taking the observed distribution of COG categories for *Blattabacterium* strain Bge as the expected distribution followed by each of the other bacteria examined, the hypothesis of equal distribution was rejected in all but the carpenter ant endosymbionts, Gamma-Proteobacteria *B. floridanus* and *B. pennsylvanicus* (Table 2). These results suggest that it is the hosts’ diet (cockroaches and carpenter ants are both omnivores) rather than phylogenetic closeness which is more strongly linked with the type of genes retained. This appears to be a clear case of functional evolutionary convergence in a broad sense. The proximity between the endosymbionts from omnivorous hosts was also confirmed when a dendrogram was created using the matrix of Kulczynski phenetic distances (Figure 3A). To locate the phylogenetic position of *Blattabacterium* strain Bge and compare it with the COG-based functional analysis, we used a phylogenetic tree based on 16S rDNA gene sequences (Figure 3B). As expected, the 16S rDNA gene analysis clearly separate Bacteroidetes from Proteobacteria phyla. *Blattabacterium* strain Bge clusters monophyletically within the Bacteroidetes phylum. The functional clustering differs clearly from the phylogenetic one.

**Table 2:** Observed COG distribution in nine bacterial genomes.

COG	Flavobacteria					Proteobacteria							
	BLB	SMU	FPS	FJO	GFO	BFL	BPE	BAP	BCC	BCI	WGL	SGL	WRI
J	109	71	142	159	156	116	114	120	114	118	107	151	48
K	16	5	93	309	147	21	22	19	17	23	22	176	18
L	31	8	97	171	141	31	31	42	30	41	30	141	120
D	10	3	16	18	16	13	13	11	7	13	13	26	9
M	35	5	168	265	210	53	58	30	5	35	58	151	28
N	2	0	5	10	4	0	0	24	11	0	31	65	1
O	31	14	83	129	98	36	38	39	27	38	38	91	34
P	16	2	85	212	147	28	28	21	6	28	18	112	30
T	2	0	56	202	103	4	4	5	3	6	3	78	6
U	15	6	29	58	33	17	18	24	18	14	28	83	18
V	6	0	39	67	48	2	3	4	2	3	7	23	14
C	44	22	93	166	136	48	50	47	30	42	36	102	47
E	74	46	146	255	216	74	75	61	42	53	34	188	41
F	32	3	59	71	65	25	25	29	7	37	39	60	28
G	20	3	62	238	131	34	34	34	18	28	22	129	30
H	49	10	98	130	104	45	51	36	7	61	65	95	26
I	21	2	70	132	96	26	30	16	8	30	29	47	16
Q	6	2	33	107	42	3	3	4	3	4	4	31	6
R	43	8	234	442	347	35	41	36	14	33	40	259	67
S	15	0	105	295	206	21	21	17	11	21	20	200	26
Chi2 Statistic		78.98	924.34	5275.14	2166.70	38.59	38.19	259.89	174.48	43.91	430.46	2128.25	353.29
df		19	19	19	19	19	19	19	19	19	19	19	19
Chi2 p-value		2,79E-9	7,30E-184	0	0	0,0050	0,0056	3,05E-44	3,77E-27	0,0010	1,99E-79	0	2,13E-63
MC p-value		5,00E-5	5,00E-5	5,00E-5	5,00E-5	0,0066	0,0059	5,00E-5	5,00E-05	0,0020	5,00E-5	5,00E-5	5,00E-5-
Distribution like BLB ? P<0.0042		rejected	rejected	rejected	rejected	not rejected	not rejected	rejected	rejected	rejected	rejected	rejected	rejected

A chi-square test was carried out with respect to the expected distribution of *Blattabacterium strain Bge*. The COG categories and species abbreviations are as in Figure 2.

### Nitrogen economy of *Blattabacterium* strain Bge

**Figure 4:**
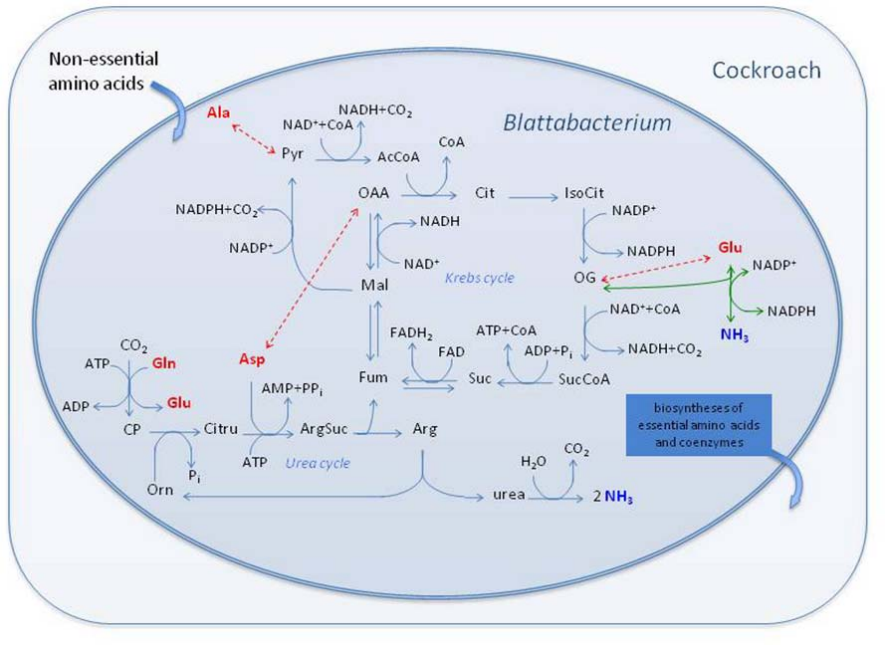
Central metabolic pathways of *Blattabacterium* strain Bge directly involved in the catabolism of amino acids. The set of balanced reactions in this diagram constitutes the input file for the stoichiometric analysis using METATOOL. Broken double arrows (in red) indicate transamination reactions. Green arrows represent the oxidative deamination of Glu. Non-conventional abbreviations: Pyr, pyruvate; AcCoA, acetyl CoA; OAA, oxalacetate; Cit, citrate; IsoCit, isocitrate; OG, 2-oxoglutarate; SucCoA, succinyl CoA; Suc, succinate; Fum, fumarate; Mal, malate; CP, carbamoyl phosphate; Citru, citrulline; ArgSuc, argininosuccinate.

A striking trait of this genome is the presence of a complete urea cycle (Figure 4). This feature has been described in few bacteria, and in only one member of the *Bacteroidetes* phylum, the cellulolytic soil bacterium *Cytophaga hutchinsonii* [25]. Moreover, to date, there are no reports of a complete urea cycle in an endosymbiont. The *Blattabacterium* strain Bge genome also retains the genes for the catalytic core of urease and we have detected urease activity in endosymbiont-enriched extracts of cockroach fat body (see below).

The genome of *Blattabacterium* strain Bge has two urease genes, *ure*AB and *ure*C, coding for the catalytic subunits, but lacks all genes for the accessory proteins supposedly required to produce an active enzyme in most bacteria. The *ure*AB fusion is not a novel situation since fused urease genes have also been described in other bacterial genomes, as it is the case of the free-living Flavobacterium *C. hutchinsonii* [25]. Regarding the lack of accessory genes, a similar situation is found in *Bacillus subtilis* cells expressing urease activity, which are able to grow with urea as sole nitrogen source [26]. To corroborate the presence of an active urease in *Blattabacterium* strain Bge, we performed an enzymatic assay on crude extracts of the endosymbiont-enriched fraction of the *B. germanica* fat body. Figure S4 shows a representative result for the urease assay. Although the detected specific activity under our experimental conditions was low (2 mU mg^−1^ protein; 1 U of urease corresponds to the formation of 1 µmol of ammonia per min), it was reproducible. Urease activity was also reproducibly detected in endosymbiont extracts from *P. americana* fat body (data not shown).

To further study the inferred metabolism in relation to nitrogen economy, we carried out a stoichiometric analysis of the reactions involved in the Krebs and urea cycles as well as other directly related reactions, such as urease, the malic enzyme, and their links to amino acid utilization (Figure 1 and Figure 4). Our results strongly suggest a key involvement of the endosymbionts in nitrogen metabolism and excretion in the German cockroach, in addition to their role in providing essential amino acids and coenzymes to the host. It is also worth mentioning that the endosymbiont metabolism relies on a supply of Gln from the host to cater for all its biosynthetic needs, including the urea cycle. Stoichiometric analysis shows that eleven out of fourteen elementary modes produce ammonia (Table S1). It follows that the metabolic network of *Blattabacterium* strain Bge could potentially use amino acids efficiently as energy and reducing-power sources, generating nitrogen waste in the form of ammonia (Figure 4).

### Comparison of nitrogen economy in endosymbionts of omnivorous hosts

Urease genes are also present in the *Blochmannia* endosymbiont genome [15] and the biochemical function of the urease in the carpenter ant endosymbionts is completely different from *Blattabacterium*. Studies of gene expression [27] and feeding experiments with ^15^N-labelled urea [17] in carpenter ants corroborate the role of urease in the transfer of nitrogen from dietary urea into the hemolymph amino acid pool. This requires an endosymbiont glutamine synthase to act as an essential step in nitrogen conservation during amino acid anabolism. Thus, although carpenter ants are omnivorous, their bacterial endosymbionts may upgrade their diet via an efficient nitrogen economy [17]. German cockroaches are also omnivorous; however, their endosymbionts lack genes encoding a glutamine synthase-like activity, a clear indication that the metabolic function of urease is not the same in the German cockroach and carpenter ant endosymbionts because generated ammonia cannot be re-assimilated. Therefore, although we have revealed a functional convergence between the cockroach and carpenter ant endosymbionts, which is probably due to their hosts’ omnivorous diets, they differ greatly from a metabolic viewpoint in detail, particularly in terms of nitrogen metabolism.

Traditionally, *Blattabacterium* endosymbionts have been postulated to be involved in the metabolism of uric acid in cockroaches. For instance, uric acid accumulation has been observed in aposymbiotic cockroaches [28],[29]. Metabolic use of nitrogen derived from fat body urates has been observed in *B. germanica* under certain conditions (e.g., in females on low-protein diet [30] and consumption of empty spermatophores by starved females [31]). Interestingly, fat body endosymbionts have been involved in uric acid degradation to CO_2_ in experiments with the wood cockroach *Parcoblatta fulvescens* injected with ^14^C-hypoxanthine [32]. Although involvement of gut microbiota cannot be completely ruled out, endosymbiont metabolism seemed more likely [33]. However, our results show that the endosymbiont genome does not code for any activity related to either the synthesis or the catabolism of urates. Therefore, and contrary to early reports based on putative cultured endosymbiotic bacteria [29], *Blattabacterium* strain Bge cannot participate in the metabolism of this nitrogen compound directly. Since uricase activity has been detected in the fat body of the cockroach [28],[34],[35], the host could contribute with uric-derived metabolites to the nitrogen economy of the endosymbiont which, in turn, would produce ammonia and carbon dioxide as final catabolic products.

### The question of ammonotelism

The genome sequencing, metabolic inference, detection of a urease in the endosymbiont and the stoichiometric analysis of the central pathways of *Blattabacterium* strain Bge shed light on a whole series of hitherto unexplained classical physiological studies on ammonotelism in cockroaches [33],[36],[37]. Contrary to the speculation that some terrestrial invertebrates, like gastropods, annelids [36] and isopods [38], exploit ammonia excretion as “a return to the cheapest way” [38] to eliminate nitrogen, the case of the German cockroach and its bacterial endosymbionts indicates that this might not be the case. The evolution of terrestrial-living metazoa has favored the emergence of uricotely (e.g. the majority of insects) and ureotely (e.g. mammals) as water-saving strategies. Meanwhile, ammonotely, the ancestral character present in aquatic animals, has classically been considered maladaptive for terrestrial animals [18]. Symbiosis seems to play a role in this “return” of cockroaches to ammonotely by providing new enzymes required for this new nitrogen metabolism. Thus the metabolic capabilities acquired by symbiogenesis [39] afford to explore new ecological niches and dietary regimes.

## Materials and Methods

### *Blattabacterium* strain Bge genomic DNA preparation

*B. germanica* (Blattaria: Blattellidae) was reared in the Entomology laboratory (Cavanilles Institute for Biodiversity and Evolutionary Biology, University of Valencia). The cockroaches were kept in the laboratory at 25°C and fed with a mixture of dog food (2/3) and sucrose (1/3).

The bacterial endosymbionts were extracted from the fat body of *B. germanica* females. To do so, cockroaches were killed by a 15 to 20 min treatment with ethyl acetate and the bacterial cells were separated from the fat body as in [15]. An enriched fraction of bacteriocytes is then obtained that is used to extract total DNA following a CTAB (Cetyltrimethylammonium bromide) method.

### Sequencing of *Blattabacterium* strain Bge genome

The complete genome sequence of *Blattabacterium* strain Bge was obtained by a hybrid sequencing approach based on ABI 3730 sequencers and the pyrosequencing system (454; Life Science). To construct shotgun libraries, DNA fragments were generated by random mechanical shearing with a sonicator and posterior separation in a pulsed field gel electrophoresis. Insert sizes of 1–2 kb and 3–5 kb were purified and cloned into vector from XL-TOPO PCR cloning kit. Plasmid DNA was extracted using 96-well plates (Millipore) with the PerkinElmer MULTIPROBE II robot according to the manufacturers. DNA sequencing was performed on an ABI PRISM 3730 Genetic Analyzer (Applied Biosystems). In the initial random sequencing phase 9,227 sequences were obtained with 1.5-fold sequence coverage. Given the lack of joining between sequences, which may have been due to a large number of sequences from the host, a strict sequence analysis was performed with a specific bioinformatic tool called a *Categorizer*. It carries out a sequence classification method based on n-mers composition to correctly distinguish between *Blattabacterium* strain Bge and contaminating host sequences. This classifier was trained with sets of sequences identified from *Blattabacterium* strain Bge and the host. With these sets, we constructed a feature vector or model representing the 4- to 7-mers usage pattern of each organism. Then the n-mers composition of each read was compared with these generated models with a k-nearest neighbor clustering algorithm (KNN).

Although the number of retrieved host sequence reads was higher than the one of *Blattabacterium* strain Bge sequences for both sequencing approaches, the pyrosequencing approach generated enough sequences to close the gaps identified with the first method. The tool Gap4 from Staden Package [40] was used for the total assembly.

### Electron microscopy of *Blattabacterium* strain Bge

Fat body of *B. germanica* was isolated and prefixed in a 2.5% paraglutaraldehyde fixative mixture buffered with 0.1 M phosphate at pH 7.2 (PB). Prefixation was performed at 4°C for 24 h and then rinsed several times in PB. To avoid the loss of this dispersed tissue, the fat body was placed in agar (2%) forming small blocks. After prefixation, these blocks were fixed in 2% osmium tetroxide for one hour, dehydrated in graded alcohol and propylene oxide, stained in a saturated uranyl acetate solution 2% and embedded in araldite to form the definitive blocks. Thin sections (0.05 µm) were made using the Reichert-Jung ULTRACUT E (Leica) ultramicrotome, and then were stained with uranyl acetate and lead citrate. A JEOL-JEM 1010 electron microscope was used for the analysis.

### ORF prediction and gene annotation

The putative coding regions (CDSs) in the *Blattabacterium* strain Bge genome were identified with the GLIMMER3 program [41]. This program was first trained with closely related organism sequences from the Flavobacteria group. The coding sequence model obtained was then used by GLIMMER3 to scan the genome to predict potential coding regions by considering the putative existence of initiation codons and ORF length. Start and stop codons of each putative CDS were curated manually through visual inspection of the *Blattabacterium* strain Bge Genome Browser, a database specially designed for this symbiont. The putative coding proteins were initially analyzed by reciprocal best hits to determine orthology between genes of the *Blattabacterium* and those from bacteria belonging to the Flavobacteria group. According to these criteria, two genes are orthologs when a gene in one genome matches as the best hit with a gene in the other genome. Sequences that could not be assigned to any function in comparison with flavobacterial genomes were identified by searching a non-redundant protein database using BLASTX [42]. Final annotation was performed using BLASTP comparison with proteins in the NCBI and Pfam domains identified using the Sanger Centre Pfam search website. Non-coding RNAs were identified by different approaches. The tRNAscan program was used to predict tRNAs, as well as other small RNAs, like tmRNA, the RNA component of the RNase P. Signal Recognition Particle RNA were identified by programs like ARAGORN, BRUCE and SRPscan, as well as consulting the Rfam database [43]–[45].

In the absence of a diagnostic cluster of DnaA boxes, the origin of replication was identified by GC-skew calculated as (C−G)/(C+G) using the program OriginX [46]. The origin is located in the transitional region where the GC-skew changes from negative to positive values.

### Inferred metabolism of *Blattabacterium* strain Bge

The ORFs orthologous to known genes in other species were catalogued based on non-redundant classification schemes, such as COG (Clusters of Orthologous Groups of Proteins). A metabolic network was reconstructed using the automatic annotator server from KAAS-KEEG [47]. According to our genome annotation, each pathway was examined checking the BRENDA [48] and EcoCyc databases [49].

### COG categories: statistical tests

Comparison between the COGs distribution of each species with that of the *Blattabacterium* strain Bge was carried using chi-square tests. To avoid the problem of multiple testing, we applied the Bonferroni correction so that for each individual test the significance level was 0.05/12 = 0.0042. That is, if the p-value is lower than 0.0042 then the hypothesis is rejected. The first p-value corresponds to the standard chi-square test (Chi2 p-value, df = 19). Due to the asymptotic nature of this test, expected frequencies should be higher than 5. However, we might expect some frequencies with low values. To correct this situation we also performed a Monte-Carlo version of this test (MC p-value). We performed 19,999 simulations under the null hypothesis, which together with the observed Chi2 statistics constituted a set of 20,000 values. The MC p-value cannot be lower than 1/20,000 = 5.00E-5.

### Kulczynski distance matrix and dendrogram

The Kulczynski distance between species 1 and 2 is given by 1−0.5(Σ_j_min(y_1j_,y_2j_)/Σ_j_y_1j_ + Σ_j_min(y_1j_,y_2j_)/Σ_j_y_2j_) where j (from 1 to 20) refers to the corresponding normalized COG categories (from 0 to 1). The dendrogram was derived from the corresponding distance matrix by applying a complete clustering method in which the distance between clusters A and B is given by the highest distance between any two species belonging to A and B, respectively. The statistical significance of the clusters of the dendrogram was evaluated by bootstrap analysis based on 100,000 replicates.

### Phylogenetic analyses

The sequences of 16S rDNA were aligned with MAFFT (v6.240) [50] program. The positions for the phylogenetic analysis were derived by Gblocks v0.91b [51]. In total, 1530 nucleotides were selected. The phylogenetic reconstruction was carried out by maximum likelihood using the PHYML program [52]. The best evolutionary model chosen by MODELTEST [53] was a GTR + Gamma (G) + I (Proportion invariant). Bootstrap values were based on 1000 replicates.

### Urease assay

Abdominal fat bodies from dissected *B. germanica* adult females were homogenized with a Douce homogenizer adding a 50 mM HEPES buffer containing 1 mM EDTA, pH 7.5. The crude extract was centrifuged for 25 min at 6000 rpm at 4°C, and the pellet was resuspended with the homogenization buffer. The supernatant and a crude extract of cockroach heads (host tissue without endosymbionts) were used in control experiments. The resuspended pellet or bacteria-enriched fraction was treated with lysozyme (3.5 U mL^−1^) for 30 min at 4°C and sonicated for 5 sec. Urease activity was determined incubating the extract at 37°C with 110 mM urea. At different time intervals the reaction was stopped by adding 1 vol. 10% trichloroacetic acid and the produced ammonia was measured by the colorimetric Berthelot method [54] as described in [55]. The protein content was measured with a Nanodrop ND1000 equipment.

### Stoichiometric analysis

Stoichiometric analysis (using METATOOL) [56] was performed on the central pathways directly involved in amino acid catabolism, including the Krebs and urea cycles. Information about the reversibility of reactions was checked in the BRENDA database [48]. The input file for METATOOL is available upon request to the corresponding author.

### Database submission

The genome was sent to GenBank and has been assigned accession number CP001487.

## Supporting Information

Figure S1 Circular map of the *Blattabacterium* strain Bge genome. From outer to inner circles: Genome length (in bp), COG categories separately for both strands, GC content (red: % value above average of 27.1%, green: below average), GC skew (red: positive skew, blue: negative skew), and tRNA genes for both strands.
(0.50 MB TIF)

Figure S2 Determination of the origin of replication by GC skew analysis.
(0.37 MB TIF)

Figure S3 Electron microcopy of *Blattabacterium* strain Bge. Abbreviations are as follows: a, trophocytes; b, *Blattabacterium* strain Bge; c, bacteriocyte cytoplasm; white arrows, host-vacuole membrane.
(1.56 MB TIF)

Figure S4 Urease activity. Kinetics of ammonia production by endosymbiont-enriched extracts in the presence of 110 mM urea (blue) compared to the endosymbiont extract without urea (pink), 110 mM urea without extract (orange), 110 mM urea in the presence of a fat-body extract after endosymbiont sedimentation (green), and 110 mM urea in the presence of a cockroach head extract (brown). The increase of absorbance at 660 nm (A) through time (t in minutes) was lineally adjusted to A = 0.002t+0.248 (R^2^ = 0.975).
(0.33 MB TIF)

Table S1 Stoichiometric analysis. The results correspond to the stoichiometric analysis of the set of reactions represented in Figure 4. The METATOOL program calculates the stoichiometric matrix and several structural properties of the metabolic network under study. We indicate the Convex Basis (i.e., the dimension of the vectorial space in which all the system solutions can be represented) and the Elementary Modes (i.e., all the flux patterns which can be accomplished at steady state and cannot be decomposed into simpler flux distributions). Any steady-state solution can be represented as a linear combination of elements of the convex basis. In every case the balanced overall reaction and the involved enzymes are indicated.
(0.15 MB DOC)

## References

[1] Blochmann F (1887). Über das regelmässige Vorkommen von backterienähnlichen Gebilden in den Geweben und Eiern versichiedener Insekten.. Z Biol.

[2] Buchner P (1965). Endosymbiosis of Animals with Plant Microorganisms.

[3] Block RJ, Henry SM (1961). Metabolism of the sulphur amino acids and of sulphate in *Blattella germanica*.. Nature.

[4] Brooks MA (1970). Comments on the classification of intracellular symbiotes of cockroaches and a description of the species.. J Invert Pathol.

[5] Bandi C, Damiani G, Magrassi L, Grigolo A, Fani R (1994). Flavobacteria as intracellular symbionts in cockroaches.. Proc Biol Sci.

[6] Bandi C, Sironi M, Damiani G, Magrassi L, Nalepa CA (1995). The establishment of intracellular symbiosis in an ancestor of cockroaches and termites.. Proc Biol Sci.

[7] Clark JW, Kambhampati S (2003). Phylogenetic analysis of *Blattabacterium*, endosymbiotic bacteria from the wood roach, *Cryptocercus* (Blattodea: Cryptocercidae), including a description of three new species.. Mol Phylogenet Evol.

[8] Lo N, Bandi C, Watanabe H, Nalepa C, Beninati T (2003). Evidence for cocladogenesis between diverse dictyopteran lineages and their intracellular endosymbionts.. Mol Biol Evol.

[9] Lopez-Sanchez MJ, Neef A, Patino-Navarrete R, Navarro L, Jimenez R (2008). Blattabacteria, the endosymbionts of cockroaches, have small genome sizes and high genome copy numbers.. Environ Microbiol.

[10] McCutcheon JP, Moran NA (2007). Parallel genomic evolution and metabolic interdependence in an ancient symbiosis.. Proc Natl Acad Sci U S A.

[11] Moya A, Pereto J, Gil R, Latorre A (2008). Learning how to live together: genomic insights into prokaryote-animal symbioses.. Nat Rev Genet.

[12] Wu D, Daugherty SC, Van Aken SE, Pai GH, Watkins KL (2006). Metabolic complementarity and genomics of the dual bacterial symbiosis of sharpshooters.. PLoS Biol.

[13] Gosalbes MJ, Lamelas A, Moya A, Latorre A (2008). The striking case of tryptophan provision in the cedar aphid *Cinara cedri*.. J Bacteriol.

[14] Perez-Brocal V, Gil R, Ramos S, Lamelas A, Postigo M (2006). A small microbial genome: The end of a long symbiotic relationship?. Science.

[15] Gil R, Silva FJ, Zientz E, Delmotte F, Gonzalez-Candelas F (2003). The genome sequence of *Blochmannia floridanus*: Comparative analysis of reduced genomes.. Proc Natl Acad Sci U S A.

[16] Degnan PH, Lazarus AB, Wernegreen JJ (2005). Genome sequence of *Blochmannia pennsylvanicus* indicates parallel evolutionary trends among bacterial mutualists of insects.. Genome Res.

[17] Feldhaar H, Straka J, Krischke M, Berthold K, Stoll S (2007). Nutritional upgrading for omnivorous carpenter ants by the endosymbiont *Blochmannia*.. BMC Biol.

[18] Needham J (1938). Contributions of chemical physiology to the problem of reversibility in evolution.. Biol Rev.

[19] Nakabachi A, Yamashita A, Toh H, Ishikawa H, Dunbar H (2006). The 160-kilobase genome of the bacterial endosymbiont *Carsonella*.. Science.

[20] Duchaud E, Boussaha M, Loux V, Bernardet JF, Michel C (2007). Complete genome sequence of the fish pathogen *Flavobacterium psychrophilum*.. Nat Biotechnol.

[21] Bauer M, Kube M, Teeling H, Richter M, Lombardot T (2006). Whole genome analysis of the marine Bacteroidetes *Gramella forsetii* reveals adaptations to degradation of polymeric organic matter.. Environ Microbiol.

[22] Pace NR, Smith D (1990). Ribonuclease P: function and variation.. J Biol Chem.

[23] Kogoma T (1997). Stable DNA replication: interplay between DNA replication, homologous recombination, and transcription.. Microbiol Mol Biol Rev.

[24] Zientz E, Dandekar T, Gross R (2004). Metabolic interdependence of obligate intracellular bacteria and their insect hosts.. Microbiol Mol Biol Rev.

[25] Xie G, Bruce DC, Challacombe JF, Chertkov O, Detter JC (2007). Genome sequence of the cellulolytic gliding bacterium *Cytophaga hutchinsonii*.. Appl Environ Microbiol.

[26] Kim JK, Mulrooney SB, Hausinger RP (2005). Biosynthesis of active *Bacillus subtilis* urease in the absence of known urease accessory proteins.. J Bacteriol.

[27] Zientz E, Beyaert I, Gross R, Feldhaar H (2006). Relevance of the endosymbiosis of *Blochmannia floridanus* and carpenter ants at different stages of the life cycle of the host.. Appl Environ Microbiol.

[28] Pierre L (1964). Uricase activity of isolated symbionts and the aposymbiotic fat body of a cockroach.. Nature.

[29] Donnellan JF, Kilby BA (1967). Uric acid by symbiotic bacteria from the fat body of *Periplaneta americana*.. Comp Biochem Physiol.

[30] Mullins DE, Keil CB, White RH (1992). Maternal and paternal nitrogen investment in *Blattella germanica* (L.) (Dictyoptera; Blattellidae).. J Exp Biol.

[31] Mullins DE, Keil CB (1980). Paternal investment of urates in cockroaches.. Nature.

[32] Cochran DG, Mullins DE (1982). Physiological processes related to nitrogen excretion in cockroaches.. J Exp Zool.

[33] Cochran DG (1985). Nitrogen excretion in cockroaches.. Annu Rev Entomol.

[34] Cordero SM, Ludwig D (1963). Purification and activities of puryne enzymes from various tissues of the American cockroach *Periplaneta americana* (L.).. J N Y Entomol Soc.

[35] Lisa JD, Ludwig D (1959). Uricase, guanase, and xanthine oxidase from the fat body of the cockroach, *Leucophaea maderae*.. Ann Entomol Soc Am.

[36] O'Donnell M (2008). Insect excretory mechanisms.. Advances in Insect Physiology.

[37] Mullins DE, Cochran DG (1972). Nitrogen excretion in cockroaches: uric acid is not a major product.. Science.

[38] Wieser W (1972). A glutaminase in the body wall of terrestial isopods.. Nature.

[39] Margulis L (1993). Symbiosis in Cell Evolution. Microbial Communities in the Archaean and Proterozoic Eons.

[40] Staden R, Beal KF, Bonfield JK (2000). The Staden package, 1998.. Methods Mol Biol.

[41] Delcher AL, Harmon D, Kasif S, White O, Salzberg SL (1999). Improved microbial gene identification with GLIMMER.. Nucleic Acids Res.

[42] Altschul SF, Madden TL, Schaffer AA, Zhang J, Zhang Z (1997). Gapped BLAST and PSI-BLAST: a new generation of protein database search programs.. Nucleic Acids Res.

[43] Regalia M, Rosenblad MA, Samuelsson T (2002). Prediction of signal recognition particle RNA genes.. Nucleic Acids Res.

[44] Laslett D, Canback B, Andersson S (2002). BRUCE: a program for the detection of transfer-messenger RNA genes in nucleotide sequences.. Nucleic Acids Res.

[45] Laslett D, Canback B (2004). ARAGORN, a program to detect tRNA genes and tmRNA genes in nucleotide sequences.. Nucleic Acids Res.

[46] Worning P, Jensen LJ, Hallin PF, Staerfeldt HH, Ussery DW (2006). Origin of replication in circular prokaryotic chromosomes.. Environ Microbiol.

[47] Moriya Y, Itoh M, Okuda S, Yoshizawa AC, Kanehisa M (2007). KAAS: an automatic genome annotation and pathway reconstruction server.. Nucleic Acids Res.

[48] Chang A, Scheer M, Grote A, Schomburg I, Schomburg D (2009). BRENDA, AMENDA and FRENDA the enzyme information system: new content and tools in 2009.. Nucleic Acids Res.

[49] Caspi R, Foerster H, Fulcher CA, Kaipa P, Krummenacker M (2008). The MetaCyc Database of metabolic pathways and enzymes and the BioCyc collection of Pathway/Genome Databases.. Nucleic Acids Res.

[50] Katoh K, Misawa K, Kuma K, Miyata T (2002). MAFFT: a novel method for rapid multiple sequence alignment based on fast Fourier transform.. Nucleic Acids Res.

[51] Talavera G, Castresana J (2007). Improvement of phylogenies after removing divergent and ambiguously aligned blocks from protein sequence alignments.. Syst Biol.

[52] Guindon S, Gascuel O (2003). A simple, fast, and accurate algorithm to estimate large phylogenies by maximum likelihood.. Syst Biol.

[53] Posada D, Crandall KA (1998). MODELTEST: testing the model of DNA substitution.. Bioinformatics.

[54] Berthelot M (1859). Violet d'aniline Rep Chim App.

[55] Richterich R (1969). Clinical Chemistry. Theory and Practice.

[56] Pfeiffer T, Sanchez-Valdenebro I, Nuno JC, Montero F, Schuster S (1999). METATOOL: for studying metabolic networks.. Bioinformatics.

