# Supplementary figures and images for: Evolutionary Convergence and Nitrogen Metabolism in Blattabacterium strain Bge, Primary Endosymbiont of the Cockroach Blattella germanica
Source: PLoS Genet. 2009 Nov 13;5(11):e1000721. doi: 10.1371/journal.pgen.1000721 (PMC2768785; doi:10.1371/journal.pgen.1000721)

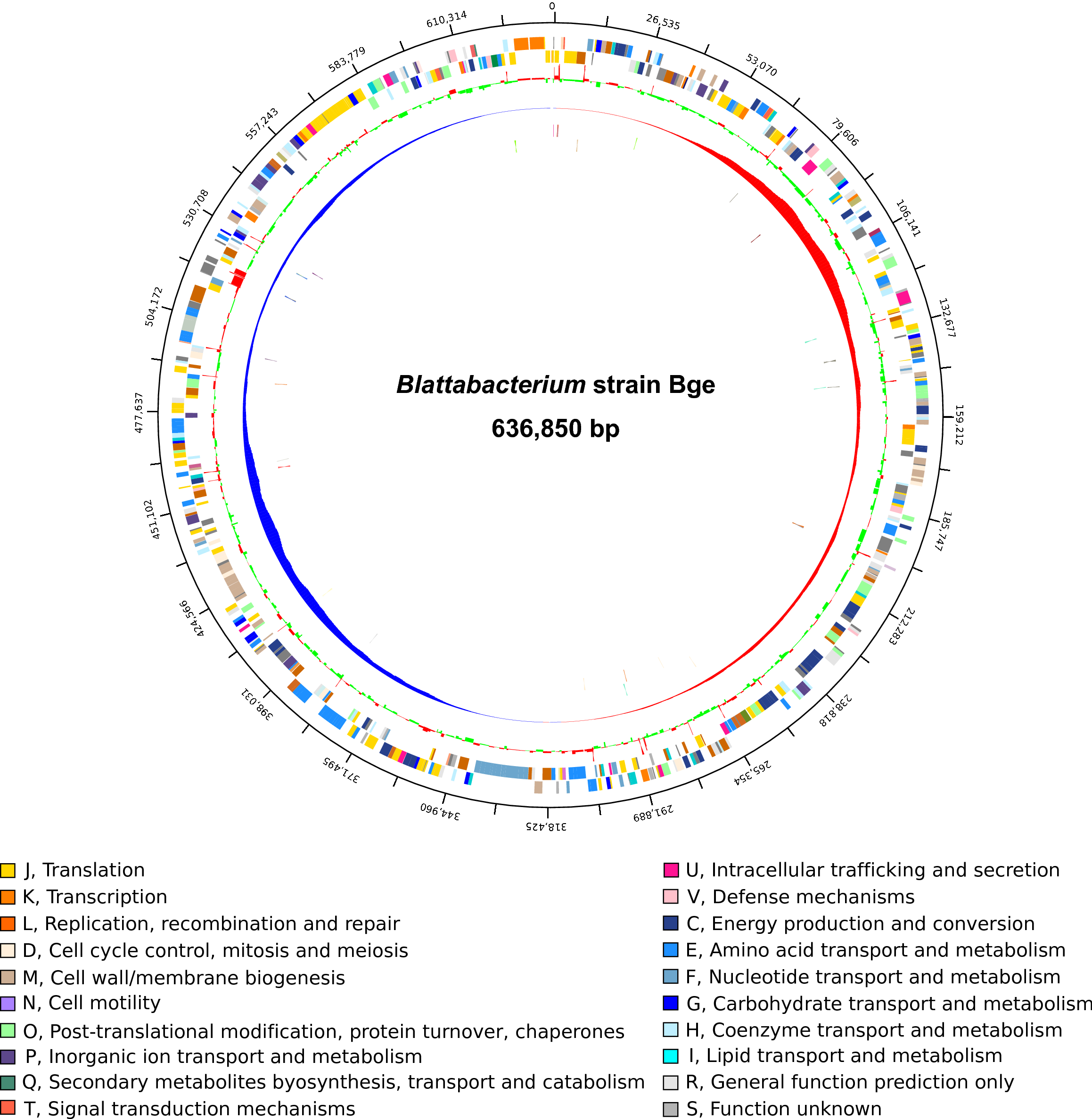

Supplement: Figure S1 — Circular map of the Blattabacterium strain Bge genome. From outer to inner circles: Genome length (in bp), COG categories separately for both strands, GC content (red: % value above average of 27.1%, green: below average), GC skew (red: positive skew, blue: negative skew), and tRNA genes for both strands. (0.50 MB TIF) [file pgen.1000721.s001.tif]

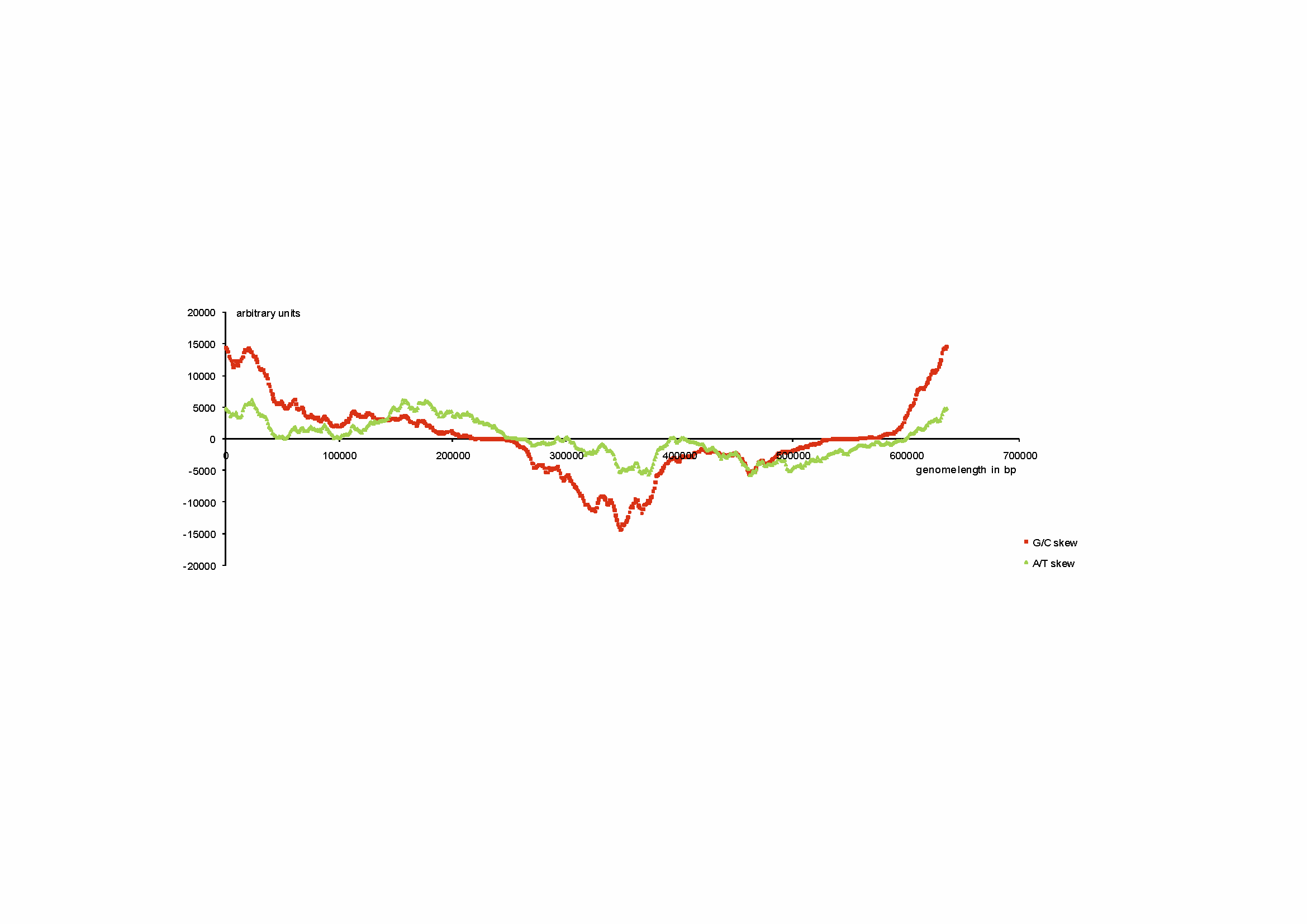

Supplement: Figure S2 — Determination of the origin of replication by GC skew analysis. (0.37 MB TIF) [file pgen.1000721.s002.tif]

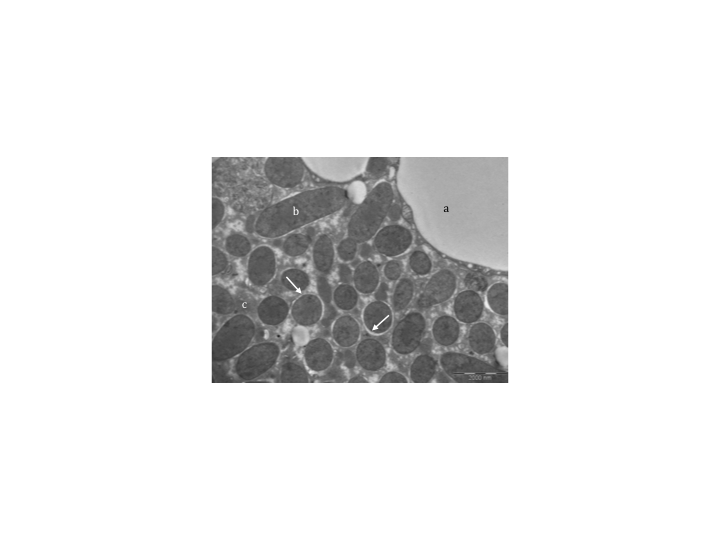

Supplement: Figure S3 — Electron microcopy of Blattabacterium strain Bge. Abbreviations are as follows: a, trophocytes; b, Blattabacterium strain Bge; c, bacteriocyte cytoplasm; white arrows, host-vacuole membrane. (1.56 MB TIF) [file pgen.1000721.s003.tif]

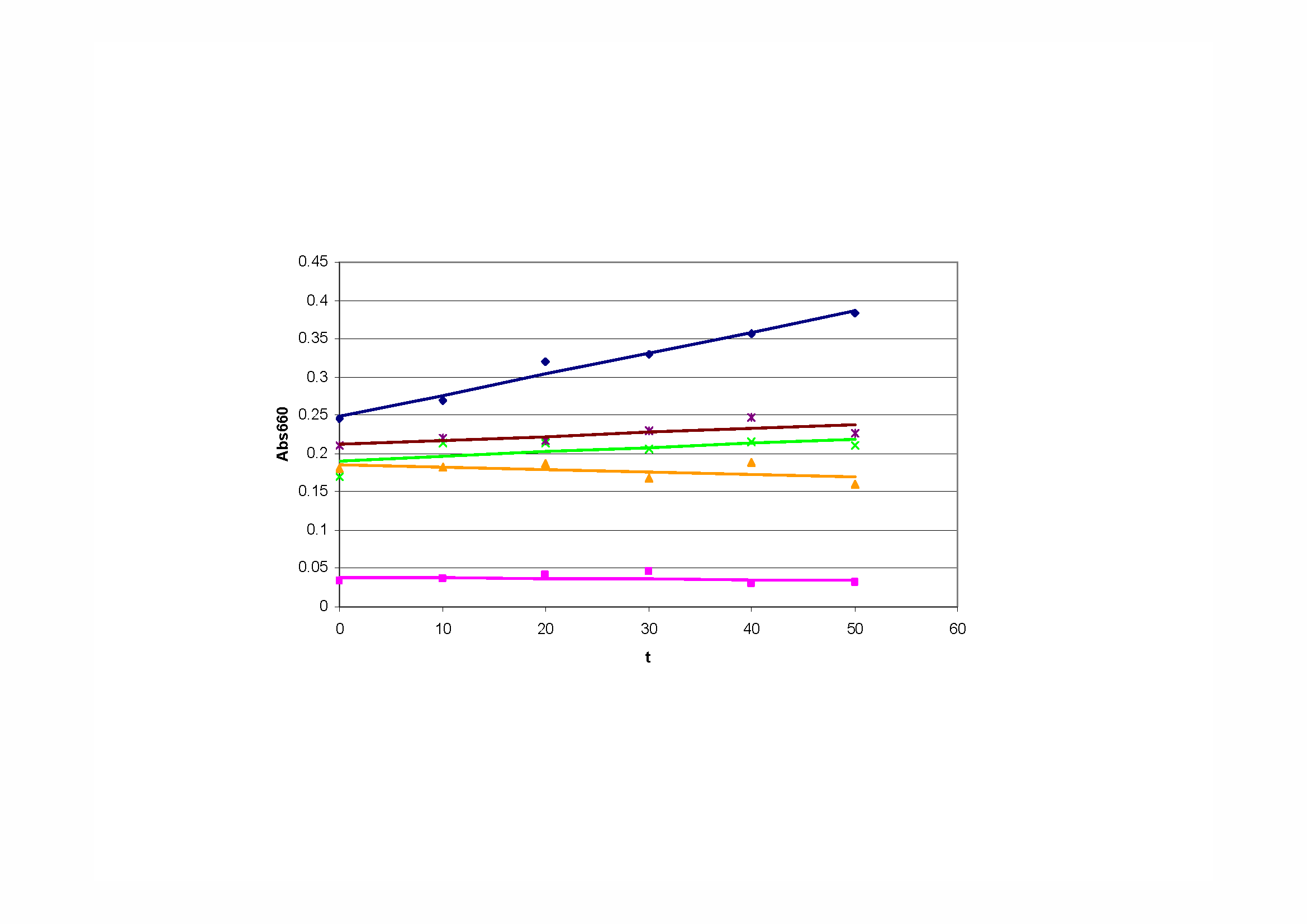

Supplement: Figure S4 — Urease activity. Kinetics of ammonia production by endosymbiont-enriched extracts in the presence of 110 mM urea (blue) compared to the endosymbiont extract without urea (pink), 110 mM urea without extract (orange), 110 mM urea in the presence of a fat-body extract after endosymbiont sedimentation (green), and 110 mM urea in the presence of a cockroach head extract (brown). The increase of absorbance at 660 nm (A) through time (t in minutes) was lineally adjusted to A = 0.002t+0.248 (R2 = 0.975). (0.33 MB TIF) [file pgen.1000721.s004.tif]
