# Supplementary material for: Evolutionary Convergence and Nitrogen Metabolism in Blattabacterium strain Bge, Primary Endosymbiont of the Cockroach Blattella germanica
Source: PLoS Genet. 2009 Nov 13;5(11):e1000721. doi: 10.1371/journal.pgen.1000721 (PMC2768785; doi:10.1371/journal.pgen.1000721)
Supplement: Table S1 — Stoichiometric analysis. The results correspond to the stoichiometric analysis of the set of reactions represented in Figure 4. The METATOOL program calculates the stoichiometric matrix and several structural properties of the metabolic network under study. We indicate the Convex Basis (i.e., the dimension of the vectorial space in which all the system solutions can be represented) and the Elementary Modes (i.e., all the flux patterns which can be accomplished at steady state and cannot be decomposed into simpler flux distributions). Any steady-state solution can be represented as a linear combination of elements of the convex basis. In every case the balanced overall reaction and the involved enzymes are indicated. (0.15 MB DOC) [file pgen.1000721.s005.doc]

**Table S1**

| **Convex basis** | **Overall reaction** | **Participating enzymes** | **Metabolic function** |
| --- | --- | --- | --- |
| 1 | ADP + FAD + 2 NAD + Glu + Pi = CO2 + ATP + FADH2 + 2 NADH + Asp | -AspC SucCD Sdh Fum Mdh SucAB  irreversible | Partial catabolism of Glu (*second half* Krebs cycle) |
| 2 | 2 ATP + NADPH + NAD + Gln + 3 H2O = ADP + NH3 + NADP + NADH + Glu + AMP + PPi + Pi | -AspC Fum Mdh Gdh CarAB ArgF ArgH RocF ArgG Urease  irreversible | Urea cycle coupled with urease |
| 3 | 2 NADP + NAD + Ala + Asp = 2 CO2 + NH3 + 2 NADPH + NADH + Glu | AspC Acn -Gdh -IlvE AceEF GltA Icd  irreversible | Partial catabolism of Ala and Asp (*first half* Krebs cycle) |
| 4 | NADP + NADH + Asp = CO2 + NADPH + NAD + Ala | AspC -Mdh IlvE MaeB  irreversible | Transhydrogenase dependent of Asp decarboxylation to Ala |
| **Elementary Modes** |  |  |  |
| 1 | ADP + FAD + 2 NAD + Glu + Pi = CO2 + ATP + FADH2 + 2 NADH + Asp | -AspC SucCD Sdh Fum Mdh SucAB  irreversible | Convex basis #1 |
| 2 | NADP + NADH + Asp = CO2 + NADPH + NAD + Ala | AspC -Mdh IlvE MaeB  irreversible | Convex basis #4 |
| 3* | 2 ATP + NADPH + NAD + Gln + 3 H2O = ADP + NH3 + NADP + NADH + Glu + AMP + PPi + Pi | -AspC Fum Mdh Gdh CarAB ArgF ArgH RocF ArgG Urease irreversible | Convex basis #2 |
| 4* | 2 ATP + NADP + 2 NAD + Ala + Asp + Gln + 3 H2O = ADP + 2 CO2 + 2 NH3 + NADPH + 2 NADH + 2 Glu + AMP + PPi + Pi | Acn Fum Mdh -IlvE AceEF GltA Icd CarAB ArgF ArgH RocF ArgG Urease  irreversible | Partial catabolism of Ala and Glu |
| 5 | ADP + FAD + NADP + NAD + Glu + Pi = 2 CO2 + ATP + FADH2 + NADPH + NADH + Ala | SucCD Sdh Fum IlvE SucAB MaeB  irreversible | Partial catabolism of Glu |
| 6* | 2 NADP + NAD + Ala + Asp = 2 CO2 + NH3 + 2 NADPH + NADH + Glu | AspC Acn -Gdh -IlvE AceEF GltA Icd  irreversible | Convex basis #3 |
| 7* | ADP + FAD + 2 NADP + 3 NAD + Ala + Pi = 3 CO2 + ATP + NH3 + FADH2 + 2 NADPH + 3 NADH | Acn SucCD Sdh Fum Mdh -Gdh -IlvE AceEF GltA Icd SucAB irreversible | Complete catabolism of Ala *via* Krebs cycle |
| 8* | 2 ATP + Asp + Gln + 3 H2O = ADP + CO2 + NH3 + Ala + Glu + AMP + PPi + Pi | Fum Gdh IlvE MaeB CarAB ArgF ArgH RocF ArgG Urease irreversible | Decarboxylation of Asp to Ala |
| 9* | 3 NADP + 2 Asp = 3 CO2 + NH3 + 3 NADPH + Glu | (2 AspC) Acn -Mdh -Gdh AceEF GltA Icd MaeB irreversible | Partial catabolism of Asp |
| 10* | ADP + FAD + 3 NADP + 2 NAD + Asp + Pi = 4 CO2 + ATP + NH3 + FADH2 + 3 NADPH + 2 NADH | AspC Acn SucCD Sdh Fum -Gdh AceEF GltA Icd SucAB maeB irreversible | Complete catabolism of Asp *via* Krebs cycle |
| 11* | 2 ATP + 2 NADP + NAD + 2 Asp + Gln + 3 H2O = ADP + 3 CO2 + 2 NH3 + 2 NADPH + NADH + 2 Glu + AMP + PPi + Pi | AspC Acn Fum AceEF GltA Icd MaeB CarAB ArgF ArgH RocF ArgG Urease irreversible | Catabolic combination of Krebs and urea cycles |
| 12* | ATP + FAD + 2 NADP + 3 NAD + Asp + Gln + 3 H2O = 4 CO2 + 2 NH3 + FADH2 + 2 NADPH + 3 NADH + Glu + AMP + PPi | Acn SucCD Sdh (2 Fum) Mdh AceEF GltA Icd SucAB MaeB carAB ArgF ArgH RocF ArgG Urease irreversible | Complete catabolism of Asp *via* Krebs and urea cycles |
| 13* | 4 ATP + NADP + 2 NAD + 2 Asp + 2 Gln + 6 H2O = 2 ADP + 3 CO2 + 3 NH3 + NADPH + 2 NADH + 3 Glu + 2 AMP + 2 PPi + 2 Pi | Acn (2 Fum) Mdh Gdh AceEF GltA Icd MaeB (2 CarAB) (2 ArgF) (2 ArgH) (2 RocF) (2 ArgG) (2 Urease) irreversible | Transhydrogenase dependent of decarboxylation of Asp |
| 14* | 2 ADP + 2 FAD + 3 NADP + 4 NAD + Glu + 2 Pi = 5 CO2 + 2 ATP + NH3 + 2 FADH2 + 3 NADPH + 4 NADH | Acn (2 SucCD) (2 Sdh) (2 Fum) Mdh -Gdh AceEF GltA Icd (2 SucAB) MaeB irreversible | Complete catabolism of Glu *via* Krebs cycle |

The elementary modes 4, 5, 7, 8, 9, 10, 11, 12, 13 and 14 are additional to the convex basis.

The elementary modes (*) 3, 4, 6, 7, 8, 9, 10, 11, 12, 13 and 14 are ammonia-producing.
